# pNIPAm-Based pH and Thermoresponsive Copolymer Hydrogel for Hydrophobic and Hydrophilic Drug Delivery

**DOI:** 10.3390/gels10030184

**Published:** 2024-03-07

**Authors:** Anandhu Mohan, Madhappan Santhamoorthy, Thi Tuong Vy Phan, Seong-Cheol Kim

**Affiliations:** 1 Department of Nano Science and Technology Convergence, General Graduate School, Gachon University, 1342 Seongnam-Daero, Sujeong-gu, Seongnam-si 13120, Gyeonggi-do, Republic of Korea; anandhumohan1993@gachon.ac.kr; 2 School of Chemical Engineering, Yeungnam University, Gyeongsan 38541, Gyeongbuk, Republic of Korea; santham83@yu.ac.kr; 3 Center for Advanced Chemistry, Institute of Research and Development, Duy Tan University, 03 Quang Trung, Hai Chau, Danang 550000, Vietnam; 4 Faculty of Environmental and Chemical Engineering, Duy Tan University, 03 Quang Trung, Hai Chau, Danang 550000, Vietnam

**Keywords:** dual stimuli, drug delivery, hydrophobic and hydrophilic drug, biocompatibility

## Abstract

The regulated and targeted administration of hydrophobic and hydrophilic drugs is both promising and challenging in the field of drug delivery. Developing a hydrogel which is responsive to dual stimuli is considered a promising and exciting research area of study. In this work, melamine functionalized poly-N-isopropyl acrylamide-co-glycidyl methacrylate copolymer has been developed by copolymerizing glycidyl methacrylate (GMA) monomer with N-isopropyl acrylamide (NIPAm) and further functionalized with melamine units (pNIPAm-co-pGMA-Mela). The prepared pNIPAm-co-pGMA-Mela copolymer hydrogel was characterized using various characterization techniques, including ^1^H NMR, FTIR, SEM, zeta potential, and particle size analysis. A hydrophobic drug (ibuprofen, Ibu) and hydrophilic drug (5-fluorouracil, 5-Fu) were selected as model drugs. Dual pH and temperature stimuli-responsive drug release behavior of the pNIPAm-co-pGMA-Mela hydrogel was evaluated under different pH (pH 7.4 and 4.0) and temperature (25 °C, 37 °C, and 45 °C) conditions. Furthermore, the in vitro biocompatibility of the developed pNIPAm-co-pGMA-Mela copolymer hydrogel was determined on MDA-MB-231 cells. The pH and temperature-responsive drug delivery study results reveal that the pNIPAm-co-pGMA-Mela hydrogel system is responsive to both pH and temperature stimuli and exhibits about ~100% of Ibu and 5-Fu, respectively, released at pH 4.0/45 °C. Moreover, the MTT assay and hemocompatibility analysis results proved that the pNIPAm-co-pGMA-Mela hydrogel system is biocompatible and hemocompatible, suggesting that that it could be used for drug delivery applications. The experimental results suggest that the proposed pNIPAm-co-pGMA-Mela hydrogel system is responsive to dual pH and temperature stimuli, and could be a promising drug carrier system for both hydrophilic and hydrophobic drug delivery applications.

## 1. Introduction

Sustained and targeted administration of hydrophilic or hydrophobic drugs to a specific location is an increasingly important area of research in the field of drug delivery; however, it encounters several challenges [1,2]. Various polymeric materials have been developed in the last two decades to overcome challenges in the drug delivery field. Block copolymers have been identified as an effective drug carrier among a variety of developed polymeric materials [3,4]. Block copolymers are a macromolecular assembly consisting of hydrophobic/hydrophilic or combined hydrophobic and hydrophilic segments. Physical entrapment or chemical conjugation can be used to load drugs into the polymeric system [5]. Because the majority of drugs consist of hydrophobic functional groups, hydrophobic interactions are applicable.

Hydrogel-based polymer technology has attracted the interest of researchers due to its three-dimensional crosslinked network structure and ability to absorb more water than its dry weight [6,7]. Hydrogels are frequently used in the chemical and biological fields because of their high hydrophilic characteristics, biocompatibility, and biodegradability. Hydrogels are considered biocompatible because of their water content and resemblance to the extracellular matrix [8,9]. Most hydrogels are transformed from sol–gel state, mostly at physiological pH (pH 7.4) and body temperature (37 °C) [10,11]. Most developed hydrogels are utilized to deliver hydrophilic drugs, as the polymer matrix is hydrophilic and consequently incompatible with the delivery of hydrophobic drugs [12,13,14]. Because many pharmacological drugs are hydrophobic, this is a significant challenge in pharmaceutical therapy. As a result, the development of hydrogels capable of accommodating hydrophobic drugs and delivering them in a regulated manner is essential [15].

N-isopropyl acrylamide (NIPAm) is a negative thermoresponsive polymer that enhances solubility at lower temperatures, and has attracted great research attention [16,17]. NIPAm possesses both hydrophilic (-CO-NH-) and hydrophobic (-(CH(CH_3_)_2_) groups in its structure. The lower critical solution temperature (LCST) of NIPAm is 32 °C, which is lower than the normal human body temperature (37 °C). To create thermoresponsive polymers, NIPAm can be copolymerized with suitable comonomers (hydrophilic/hydrophobic) to produce thermoresponsive hydrogels with increased/decreased LCST [18,19]. Because of this characteristic, pNIPAm is useful for drug delivery systems in biological applications. Similarly, poly(glycidyl methacrylate) is a promising polymer having epoxy units that may be further chemically modified. Glycidyl methacrylate (GMA) is a less expensive and low-toxicity monomer that copolymerizes readily with an appropriate comonomer [20]. The reactive epoxy groups of the GMA units easily react with thiol or amine groups containing functional molecules, increasing the practical application of the resultant copolymer system. A variety of drug molecules may be efficiently loaded into the copolymer hydrogel system below the copolymer’s LCST, and the loaded drugs can be administered at a particular location by gently raising the temperature above the LCST of the specific hydrogel system [21]. Among various stimuli that have been utilized as triggers, pH stimuli are considered to be an ideal trigger for the selective release of anticancer drugs because the pH of the tumor tissues is slightly lower (6.5–7.2) than the normal cells and blood (~pH 7.4). Therefore, the pH stimuli can control the site-specific drug release based on the pH value of the surrounding environments. Sustained drug release is considered an important factor in releasing the loaded cargo in a controlled manner to achieve prolonged drug release. In this way, the reoccurrence of tumors can be controlled over a long time period. Similarly, temperature stimuli can control drug release concerning the applied external temperature conditions. Thus, the development of drug delivery systems with dual response is considered an advantage over single stimuli-responsive drug delivery systems. There are several temperature-responsive hydrogels that have been reported in the literature. For example, Thirupathi et al. reported a pH and thermoresponsive PNIPAm-co-Polyacrylamide hydrogel for controlled drug delivery to achieve long-term drug release [22]. Kim and co-workers developed temperature-responsive polymer-modified silica nanoparticles for pH and temperature stimuli-controlled drug delivery of anticancer drugs [23]. Similarly, Asadi et al. developed an N-acetylglucosamine-loaded methylcellulose hydrogel for colon drug delivery applications [24].

In this work, we demonstrate the synthesis of a copolymer hydrogel composed of pNIPAm and poly(glycidyl methacrylate) (pGMA) segments and with epoxy groups that can be further modified with melamine (Mela) molecules to create a pNIPAm-co-pGMA-Mela hydrogel system. The pNIPAm segment in the proposed system serves as a host for a hydrophobic drug (ibuprofen, Ibu) and is responsible for temperature-responsive swelling–deswelling in response to temperature stimuli. Similarly, functionalized melamine (Mela) groups serve as hydrophilic drug hosts (5-fluorouracil, 5-Fu) and are responsible for pH-stimulated drug release. Various characterization methods, including ^1^H NMR, FTIR, SEM, zeta potential, and particle size analyses, were used to examine the developed pNIPAm-co-GMA-Mela hydrogel system. The drug release efficacy of the pNIPAm-co-pGMA-Mela hydrogel system was evaluated using Ibu and 5-Fu as typical hydrophobic and hydrophilic drugs at varying respective pH (pH 7.4 and 4.0) and temperature (25 °C, 37 °C, and 45 °C) values. In addition, the pNIPAm-co-pGMA-Mela hydrogel’s hemocompatibility and in vitro biocompatibility on MDA-MB-231 cells were evaluated.

## 2. Results and Discussion

### 2.1. pNIPAm-co-pGMA and pNIPAm-co-pGMA-Mela Hydrogel Characterization

**Figure 1:**
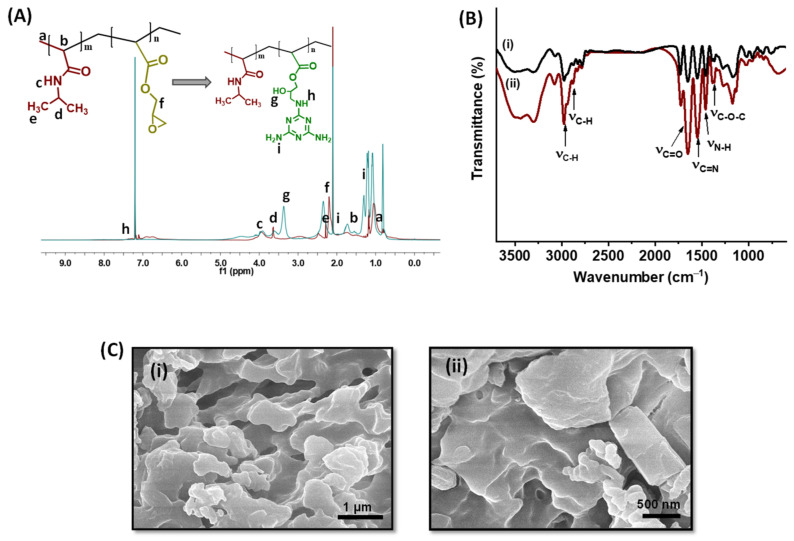
(**A**) ¹H NMR spectra of pNIPAAm-co-pGMA copolymer and pNIPAAm-co-pGMA-Mela hydrogel; (**B**) FTIR analysis of (i) pNIPAAm-co-pGMA copolymer and (ii) pNIPAAm-co-pGMA-Mela hydrogel samples; (**C**) SEM images of (**i**) pNIPAAm-co-pGMA copolymer and (**ii**) pNIPAAm-co-pGMA-Mela hydrogel system.

The synthesized pNIPAm-co-pGMA hydrogel system was characterized by ^1^H NMR spectral analysis. The ^1^H NMR spectrum of the pNIPAm-co-pGMA sample in Figure 1A shows the proton signals at δ1.22 ppm and δ2.42 ppm, indicating methyl (-CH_3_) and methoxy (-OCH_2_-), respectively. The prominent signals appearing at δ5.49 ppm and δ2.68 ppm indicate the isopropyl and amide groups, which confirms the pNIPAm-co-pGMA copolymer system composed of pNIPAm and pGMA segments. For melamine functionalization, the pNIPAm-co-pGMA-Mela hydrogel sample shows peaks at δ1.79 ppm and δ2.83 ppm for the hydroxyl (-OH) and amine (N-H) groups, respectively (Figure 1A), indicating the successful functionalization of melamine groups by the reaction of the amine part of melamine and the epoxy part of the GMA units [24,25]. Figure 1B(i,ii) displays the FTIR spectra of the pNIPAm-co-pGMA and pNIPAm-co-pGMA-Mela hydrogel system, in which the vibration bands at 2878 cm^−1^ and 2965 cm^−1^ indicate the carbon chains. The stretching peak at 1638 cm^−1^ for the carbonyl (-C=O) groups confirms the NIPAm units in the pNIPAm-co-pGMA sample [25]. Further, the vibration peak appearing at 1396 cm^−1^ for C-O-C stretching of epoxy moieties confirms the GMA units in the pNIPAm-co-pGMA sample (Figure 1B(i)). After functionalization of the Mela groups, the newly appearing peaks at 1438 cm^−1^ and 1549 cm^−1^ confirm the amine and imine groups, evidencing the presence of Mela groups in the pNIPAm-co-pGMA-Mela hydrogel system (Figure 1B(ii)) [26]. SEM analysis was performed to observe the particles and surface morphology of the powdered pNIPAm-co-pGMA and pNIPAm-co-pGMA-Mela hydrogel samples. In Figure 1C(i,ii), the pNIPAm-co-pGMA and pNIPAm-co-pGMA-Mela hydrogel samples both show microparticle arrangements with aggregation. The rough surface morphology and particle aggregation may be due to the formation of H-bonding interaction between the functionalized melamine groups and the carbonyl groups of the pNIPAm segments.

**Figure 2:**
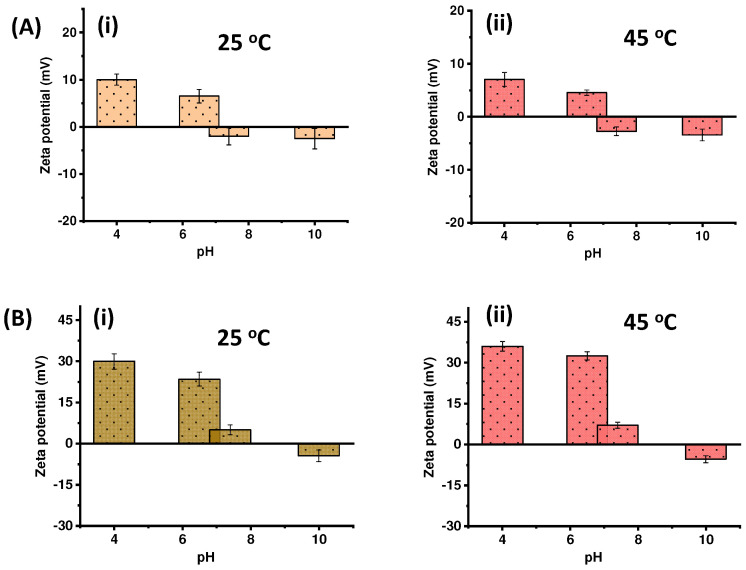
Zeta potential analysis of (**A**) pNIPAm-co-pGMA copolymer at (**i**) 25 °C and (**ii**) 45 °C and (**B**) pNIPAm-co-pGMA-Mela hydrogel at (**i**) 25 °C and (**ii**) 45 °C.

The pNIPAm-co-pGMA and pNIPAm-co-pGMA-Mela hydrogels are expected to be responsive to pH and temperature stimuli owing to their isopropyl, secondary amine, and carbonyl groups in the NIPAm segments and the free hydroxyl (-OH), amine (-NH_2_), and imine (-C=N) groups present in the melamine-functionalized GMA segments. The zeta potentials of the pNIPAm-co-pGMA and pNIPAm-co-pGMA-Mela hydrogel samples were determined at 25 °C and 45 °C. As displayed in Figure 2A(i,ii), the pNIPAm-co-pGMA sample had positive zeta potential values of +10 mV, +7.4 mV, −3.2 mV, and −2.9 mV at 25 °C and about +7 mV, 6.4 mV, −2.9 mV, and −2.2 mV at 45 °C for pH 4, 6, 8, and 10, respectively. In contrast, as shown in Figure 2B(i,ii), the pNIPAm-co-pGMA-Mela hydrogel showed higher positive zeta potential values of approximately +30 mV, +23 mV, +5 mV, and −8 mV at 25 °C, and at 45 °C the zeta potential values increased to +37 mV, +32 mV, +7.6 mV, and −10 mV at pH 4, 6, 8, and 10, respectively. This increase in positive zeta potential is due to the incorporation of melamine groups on the exposed outer surface of the copolymer chains. Under reduced acidic pH conditions, the amine parts of the melamine groups are protonated and show increased positive zeta potential at 45 °C. It is notable that the change in zeta potential value from a positive surface charge to a negative charge at >pH~7 might be due to the deprotonation of charged amine and amide groups of the pNIPAm segments and functionalized melamine groups at >pH~7. Zeta potential analysis confirmed that the melamine moieties were successfully functionalized in the synthesized pNIPAm-co-pGMA-Mela hydrogel system [27].

**Figure 3:**
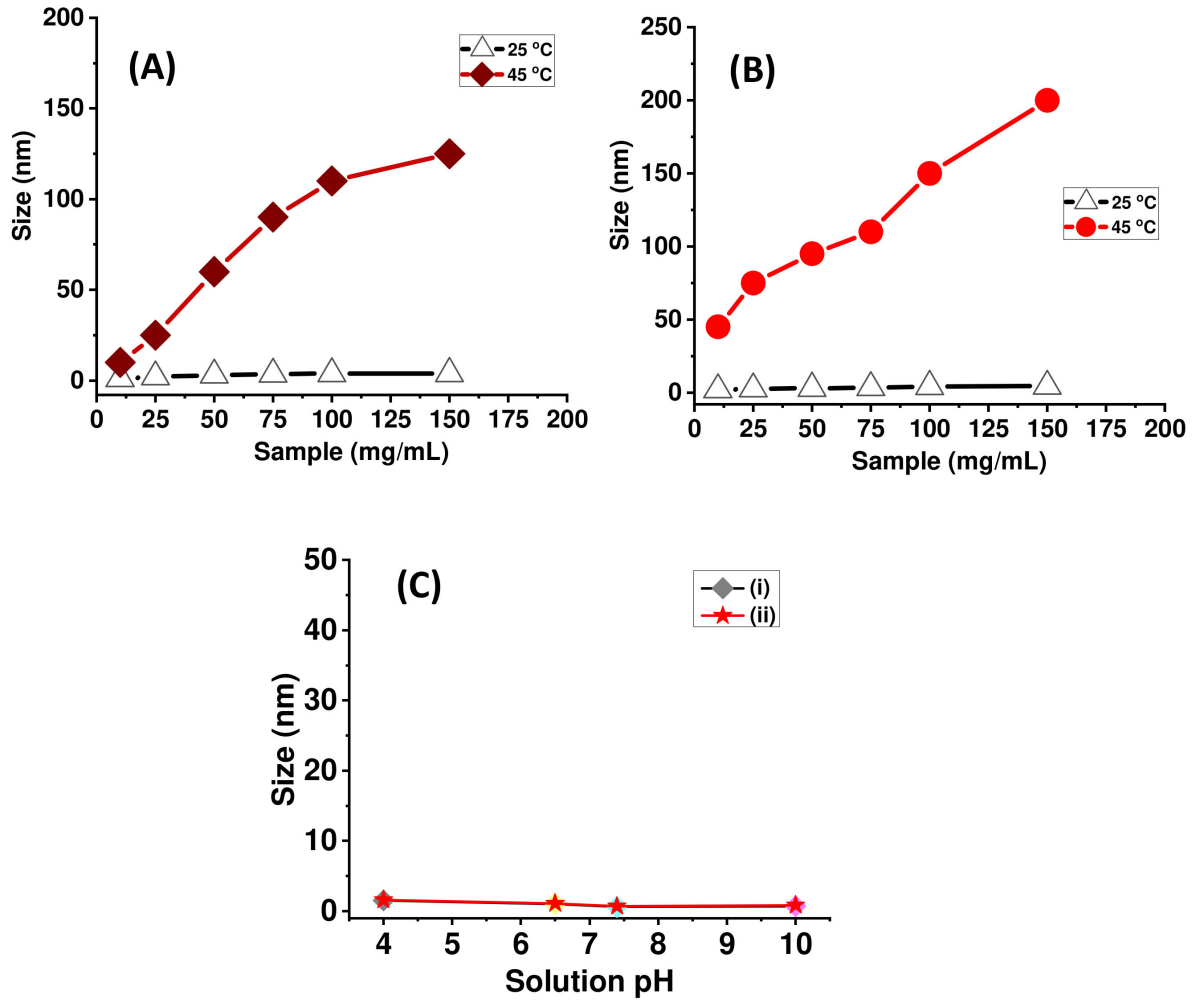
Particle size analysis of (**A**) pNIPAm-co-pGMA copolymer and (**B**) pNIPAm-co-pGMA-Mela hydrogel at different temperature conditions; (**C**) particle size analysis of (i) pNIPAm-co-pGMA copolymer and (ii) pNIPAm-co-pGMA-Mela hydrogel at different pH conditions.

The particle size of the pNIPAm-co-pGMA and pNIPAm-co-pGMA-Mela hydrogel samples were measured at 25 °C and 45 °C. As displayed in Figure 3A,B, both the pNIPAm-co-pGMA and pNIPAm-co-pGMA-Mela hydrogel samples had low particle sizes when measured at 25 °C. In contrast, the particle size was considerably increased at 45 °C. This is because the pNIPAm-co-pGMA and pNIPAm-co-pGMA-Mela hydrogels both exhibit a linear chain structure at 25 °C. On the other hand, the polymer chain structure turns into a globule structure at 45 °C due to shrinkage and phase transition. This phase transition from a linear structure to a globule structure results in increased particle size in aqueous media. Further, the larger particle size of the pNIPAm-co-pGMA-Mela hydrogel might be due to this phase transition as well as the H-bonding interaction between the particles. Similarly, the particle size analysis was carried out at different pH conditions from pH 4 to 10. As displayed in Figure 3C, the pNIPAm-co-pGMA and pNIPAm-co-pGMA-Mela hydrogel samples showed no significant particle size changes under varying pH conditions.

**Figure 4:**
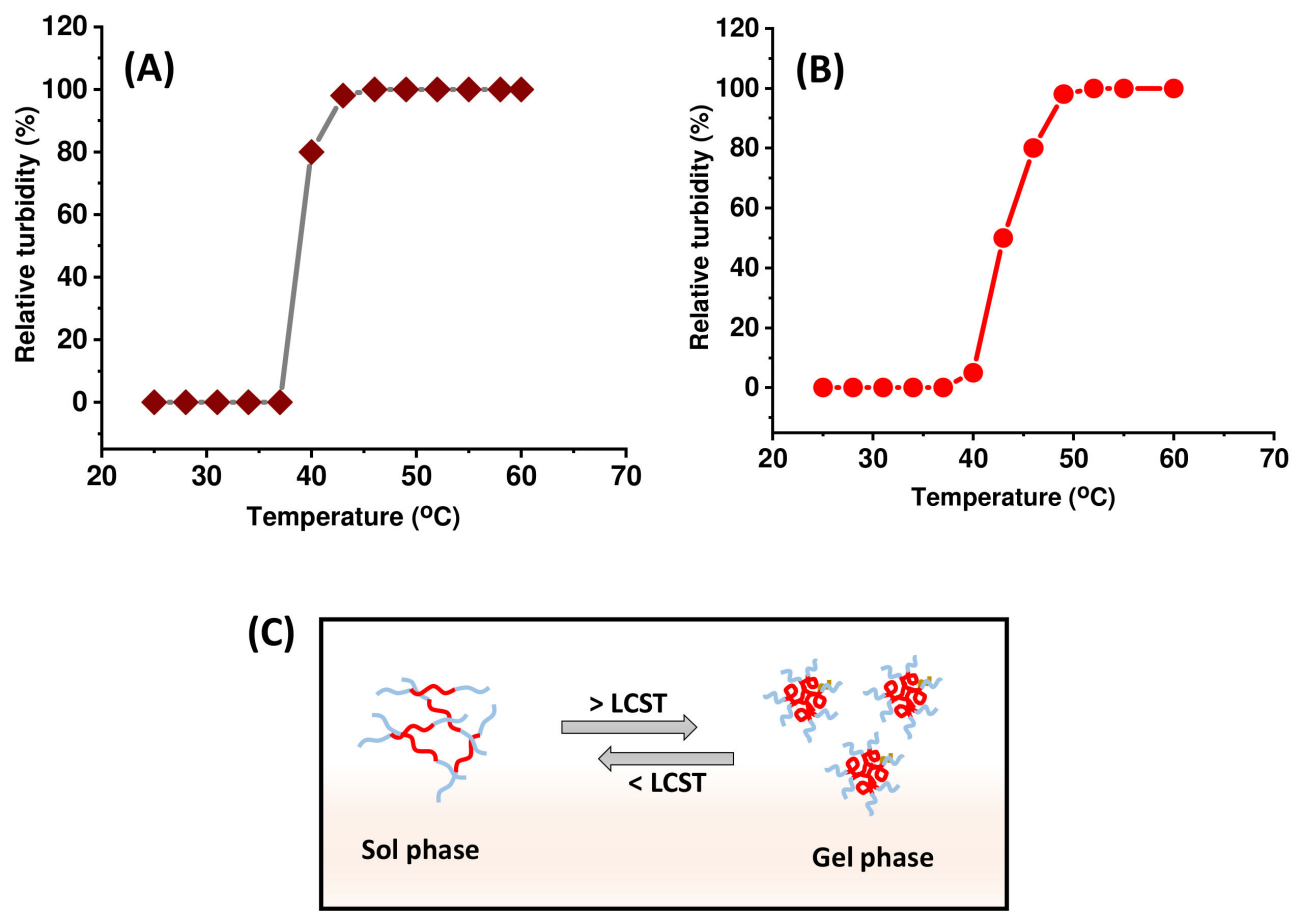
Relative turbidity of (**A**) pNIPAm-co-pGMA copolymer and (**B**) pNIPAm-co-pGMA-Mela hydrogel samples at temperatures ranging from 25 °C to 55 °C; (**C**) schematic representation of the phase transition of the pNIPAm-co-pGMA-Mela hydrogel.

The phase transition and relative turbidity of the pNIPAm-co-pGMA and pNIPAm-co-pGMA-Mela hydrogels were studied in a range of temperatures between 25 °C and 55 °C by UV–vis measurement. As displayed in Figure 4A,B, both samples showed no noticeable absorbance below 40 °C (<LCST). At <LCST, both the pNIPAm-co-pGMA and pNIPAm-co-pGMA-Mela hydrogel samples readily absorbed water and become hydrated with linear structures [28]. In contrast, the transparent polymer solution became turbid, and the solution turbidity increased when increasing the temperature above 40 °C (>LCST) (Figure 4A,B). This phase transition occurs because the hydrophilic linear polymer chains shrink to hydrophobic globular structures, as illustrated in Figure 4C [29].

**Figure 5:**
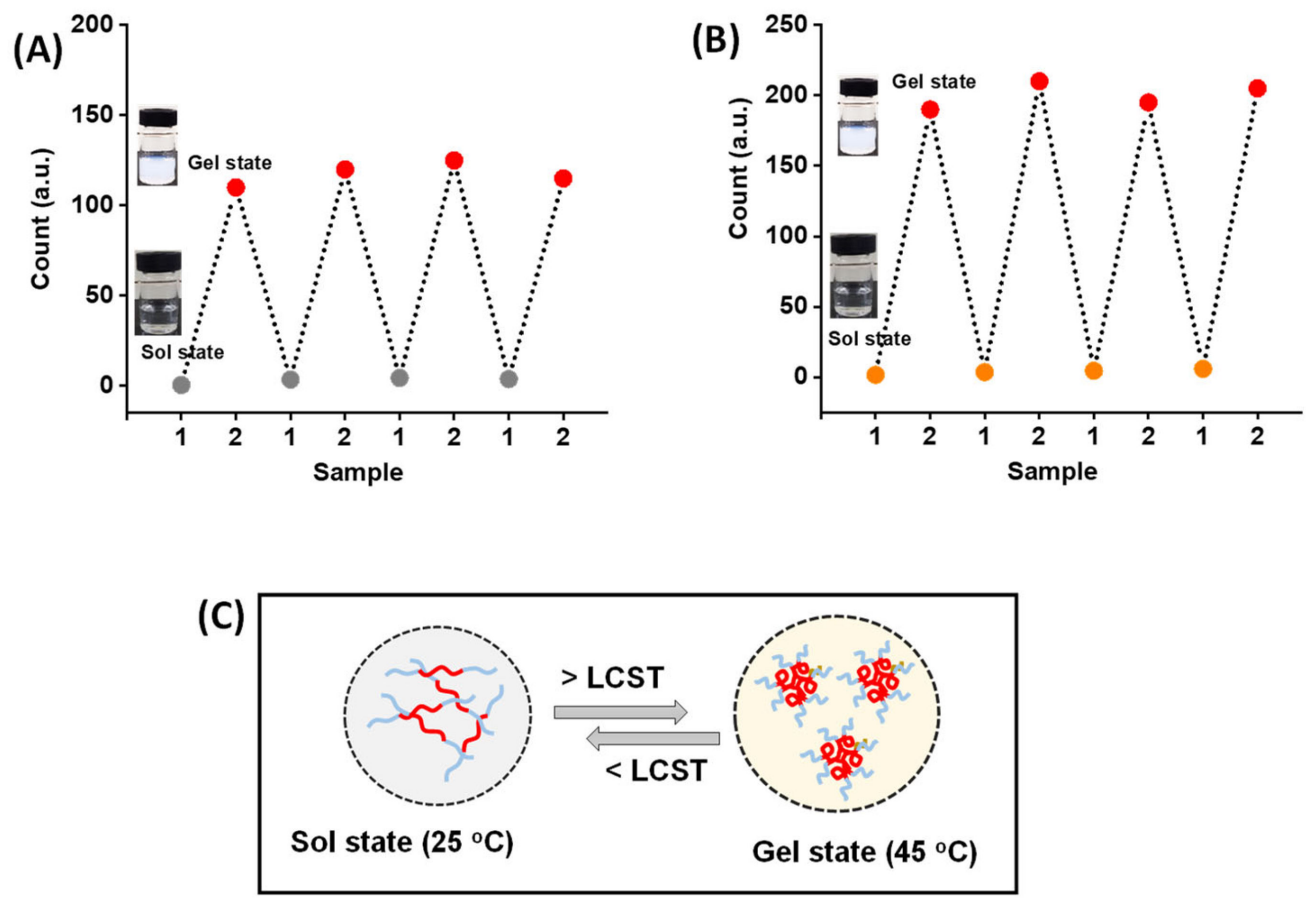
Temperature-responsive swelling-deswelling behavior of (**A**) pNIPAm-co-pGMA copolymer and (**B**) pNIPAm-co-pGMA-Mela hydrogel at temperatures ranging from25 °C to 45 °C; (**C**) schematic representation of the sol–gel phase transition of pNIPAm-co-pGMA-Mela hydrogel.

Similarly, the respective swelling–deswelling behaviors of pNIPAm-co-pGMA and pNIPAm-co-pGMA-Mela hydrogel samples were determined by DLS analysis at 25 °C and 45 °C. As displayed in Figure 5A, the pNIPAm-co-pGMA copolymer sample showed no noticeable particle size changes at 25 °C, whereas significant microparticles were formed at 45 °C. The same observation was noted for the melamine-functionalized pNIPAm-co-pGMA-Mela hydrogel sample (Figure 5B). These results suggest that the pNIPAm-co-pGMA-Mela hydrogel is thermoresponsive and undergoes a significant phase transition at the above LCST (Figure 5C) [30].

### 2.2. Stimuli-Responsive Drug Delivery Performance of pNIPAm-co-pGMA-Mela Hydrogel System

An NIPAm-based pNIPAm-co-pGMA copolymer was synthesized by combining NIPAm and GMA monomers, then was further functionalized with hydrophilic melamine groups in the side chain, resulting in a pNIPAm-co-pGMA-Mela hydrogel system. These functional groups improve the solubility of the hydrogel system. Owing to the presence of both pNIPAm (hydrophobic) and pGMA-Mela (hydrophilic) copolymer units, the pNIPAm-co-pGMA-Mela copolymer hydrogel system can produce a hydrophobic-hydrophilic domain structure in aqueous solution [31,32]. It is anticipated that the developed pNIPAm-co-pGMA-Mela hydrogel system could considerably improve drug delivery to specific sites under pH and/or temperature stimuli due to the presence of temperature-responsive pNIPAm segments and pH-responsive pGMA-Mela segments. The hydrophobic pNIPAm units in the pNIPAm-co-pGMA-Mela hydrogel can accommodate hydrophobic drugs, while the pGMA can accommodate hydrophilic drugs due to the presence of hydrophilic melamine groups in the hydrogel system [33]. The loaded drugs can then be delivered selectively by altering the pH or temperature stimuli. Therefore, the proposed pNIPAm-co-pGMA-Mela copolymer hydrogel system could be desirable for temperature- and pH-responsive drug delivery applications. To validate this, a model hydrophobic drug ibuprofen (Ibu) and 5-fluorouracil (5-Fu) was encapsulated. The Ibu and 5-Fu-loaded hydrogel samples were named pNIPAm-co-pGMA-Mela/Ibu and pNIPAm-co-pGMA-Mela/5-Fu, respectively.

**Figure 6:**
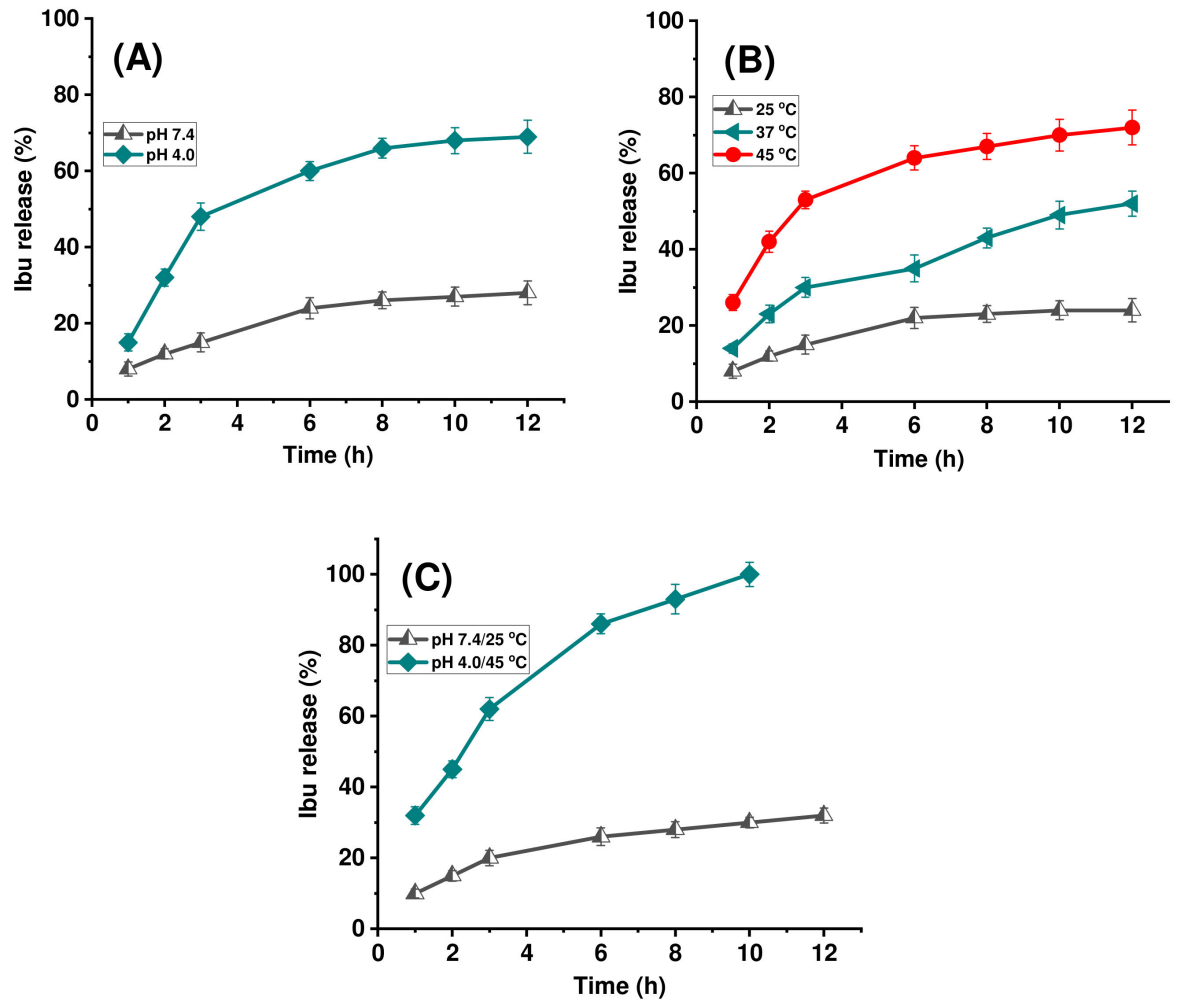
In vitro drug delivery efficiency of pNIPAAm-co-pGMA-Mela/Ibu hydrogel system: (**A**) Ibu release under various pH conditions, (**B**) Ibu release under various temperatures, and (**C**) Ibu release with different combinations of pH and temperature stimuli conditions.

The pH and temperature responsiveness were validated under various conditions over 12 h: pH (pH 7.4 and 4.0 while maintaining the solution temperature at 25 °C); temperature (25 °C, 37 °C, and 45 °C while maintaining the solution pH at 7.4); and pH + temperature (pH 7.4/45 °C and pH 4.0/45 °C). First, the release of Ibu from the Ibu-loaded pNIPAm-co-pGMA-Mela/Ibu sample was examined. Figure 6A displays the Ibu release profile from the pNIPAm-co-pGMA-Mela/Ibu hydrogel under different pH conditions. As can be observed in Figure 6A, about 27% and 69% of Ibu was released at pH 7.4 and 4.0, respectively. Similarly, approximately 18%, 42%, and 70% of Ibu was released from the pNIPAm-co-pGMA-Mela/Ibu hydrogel under the different temperature conditions (25 °C, 37°C, and 45 °C), respectively (Figure 6B). On the other hand, approximately 30% and 100% of Ibu was released under the pH 7.4/25 °C and pH 4.0/45 °C conditions, respectively (Figure 6C).

**Figure 7:**
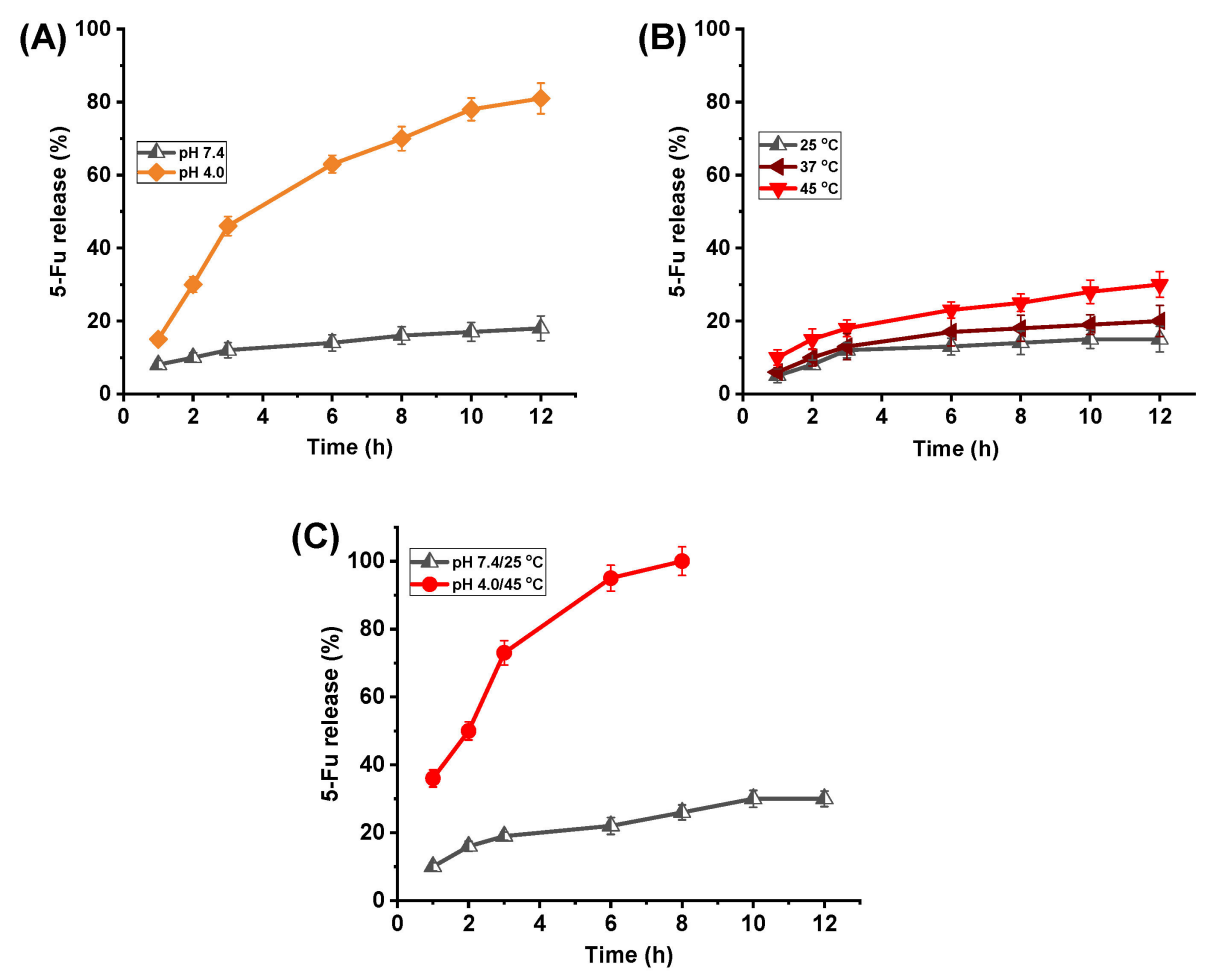
In vitro drug delivery efficiency of pNIPAAm-co-pGMA-Mela/5-Fu hydrogel system: (**A**) 5-Fu release at various pH conditions, (**B**) 5-Fu release at various temperatures, and (**C**) 5-Fu release with different combined pH and temperature conditions.

Second, the 5-Fu-loaded pNIPAm-co-pGMA-Mela/5-Fu sample was examined after 12 h while altering the pH and temperature conditions. Figure 7A shows the release of 5-Fu from the pNIPAm-co-pGMA-Mela/5-Fu hydrogel system at two different pH conditions (pH 7.4 and 4.0). As shown in Figure 7A, about 18% and 80% of 5-Fu was delivered at pH 7.4 and 4.0, respectively; furthermore, approximately 14%, 20%, and 33% of the 5-Fu was delivered from the pNIPAm-co-pGMA-Mela/5-Fu under different temperatures conditions (25 °C, 37 °C, and 45 °C), respectively (Figure 7B). In contrast, approximately 30% and 100% of 5-Fu was released under the combined pH 7.4/25 °C and pH 4.0/45 °C conditions, respectively (Figure 7C). The encapsulation of the drug in the pNIPAm-co-pGMA-Mela system was possibly achieved via electrostatic interaction and/or hydrogen bonding interactions between the Ibu and 5-Fu molecules and the drug binding sites, specifically the amine (-N-H) and (-C=O) groups present in the pNIPAm-co-pGMA-Mela copolymer system.

As observed from the Ibu and 5-Fu drug release profiles (Figure 6 and Figure 7), a higher percentage of Ibu and 5-Fu was delivered under pH 4.0 and 45 °C conditions than under pH 7.4 and 25 °C conditions. This could be due to the acidic pH-induced protonation of the secondary -N-H and -OH and primary -NH_2_ groups present in the pNIPAm-co-pGMA-Mela hydrogel [34]. Because of the protonation of the -NH, -NH_2_, and -OH groups under acidic pH circumstances, an electrostatic repulsive force occurs between the protonated drug binding sites and the protonated drug molecules, causing the loaded drug molecules to be released from the pNIPAm-co-pGMA-Mela hydrogel system [35].

Similarly, the release of Ibu or 5-Fu from the pNIPAm-co-pGMA-Mela hydrogel was higher at 45 °C than at 25 °C or 37 °C. This might be due to the temperature-triggered delivery of drug molecules from the pNIPAm-co-pGMA-Mela hydrogel system. The copolymer chains in the pNIPAm-co-pGMA-Mela hydrogel undergo a temperature-triggered polymer chain transformation from linear chains to a globular structure; therefore, the loaded drug molecules diffuse out from the pNIPAm-co-pGMA-Mela hydrogel [36]. Furthermore, the pNIPAm-co-pGMA-Mela hydrogel system showed significant drug delivery behavior at the combination of pH 4.0 and 45 °C due to the combined pH-triggered protonation and electrostatic repulsive force and temperature-induced phase transition, showing considerably enhanced drug delivery from the pNIPAm-co-pGMA-Mela hydrogel system [37]. Therefore, the pNIPAm-co-pGMA-Mela hydrogel could be utilized as a drug carrier system for hydrophobic and/or hydrophilic drug delivery applications.

### 2.3. In Vitro Biocompatibility and Hemocompatibility Study

**Figure 8:**
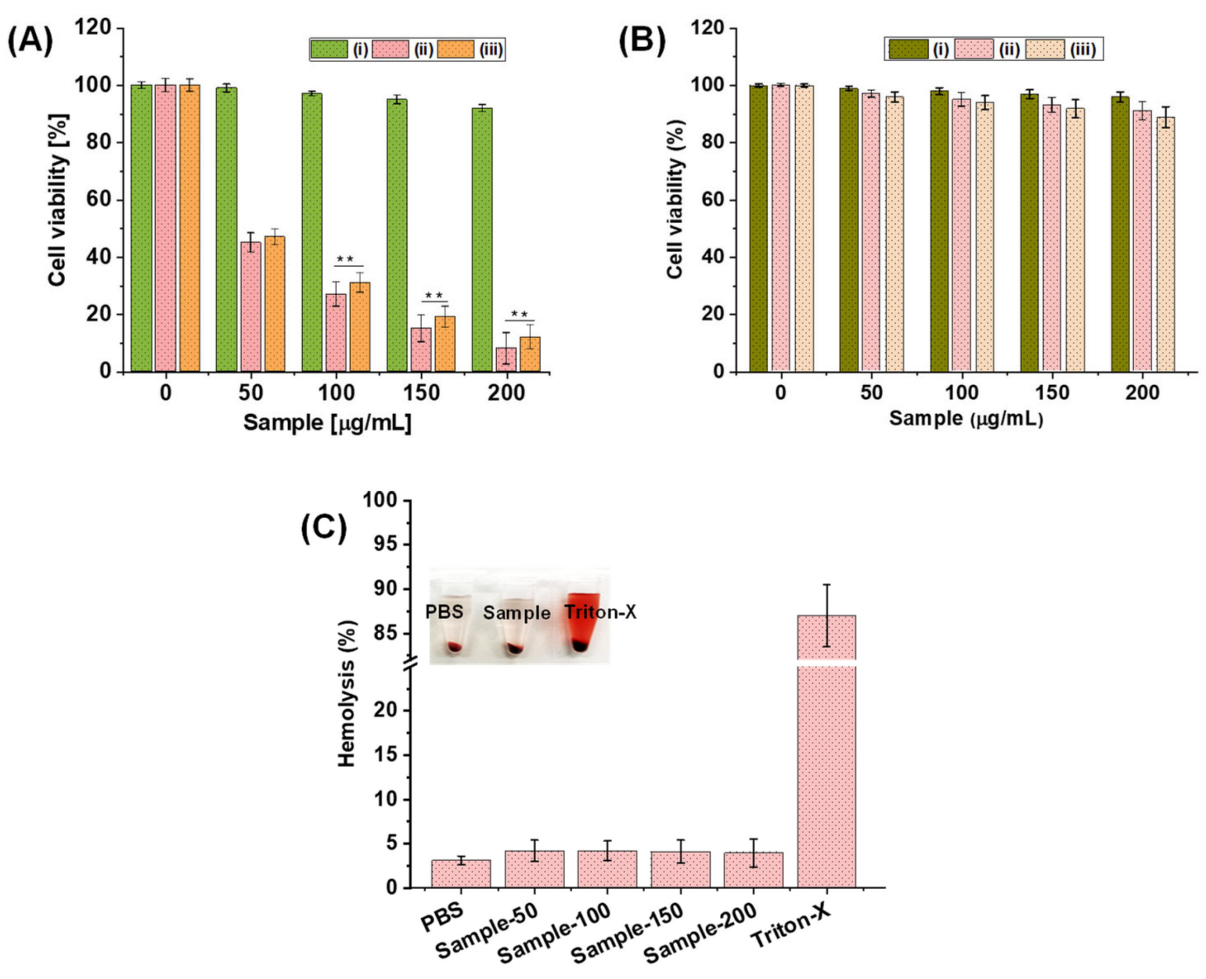
(**A**) In vitro cytocompatibility of (i) pNIPAAm-co-pGMA-Mela hydrogel, (ii) 5-Fu drug-loaded pNIPAAm-co-pGMA-Mela/5-Fu hydrogel, and (iii) pure 5-Fu drug tested on MDA-MB-231 cells at different concentrations; (**B**) in vitro cytocompatibility of (i) pNIPAAm-co-pGMA-Mela hydrogel, (ii) Ibu drug-loaded pNIPAAm-co-pGMA-Mela/Ibu hydrogel, and (iii) pure Ibu drug tested on MDA-MB-231 cells at different concentrations; and (**C**) blood compatibility behavior of pNIPAAm-co-pGMA-Mela hydrogel at different sample concentrations. The inset image shows the in vitro blood compatibility of pNIPAAm-co-pGMA-Mela hydrogel by exposure of red blood cells to PBS, pNIPAAm-co-pGMA-Mela hydrogel sample, and Triton-X. Statistical significance refers to the cell toxicity with different samples (** significant *p* < 0.05).

An MTT (3-(4,5-dimethylthiazol-2-yl)-2,5-diphenyltetrazolium bromide) assay was carried out to determine the cell compatibility of the pNIPAm-co-pGMA-Mela and 5-Fu loaded pNIPAm-co-pGMA-Mela/5-Fu hydrogel samples at different sample doses (50–200 μg/mL) using MDA-MB-231 cells. To perform this study, MDA-MB-231 cells were exposed to pNIPAm-co-pGMA-Mela and pNIPAm-co-pGMA-Mela/5-Fu hydrogel samples for 24 h. As can be observed in Figure 8A, the pNIPAm-co-pGMA-Mela hydrogel without 5-Fu loading showed about ~ 90% cell viability in the tested dosage of the sample. These results support the cytocompatibility of the pNIPAm-co-pGMA-Mela hydrogel. Moreover, the 5-Fu loaded pNIPAm-co-pGMA-Mela/5-Fu hydrogel sample and free 5-Fu drug respectively showed about 92% and 89% cell toxicity to the MDA-MB-231 cells (Figure 8A). The pNIPAm-co-pGMA-Mela/5-Fu hydrogel sample showed more cell killing ability than the pristine 5-Fu drug, as the sustained slow release of 5-Fu from the pNIPAm-co-pGMA-Mela/5-Fu hydrogel in the intracellular medium induces more cell killing than the pure 5-Fu [38]. Further, the cell viability of the Ibu-loaded pNIPAm-co-pGMA-Mela/Ibu hydrogel was evaluated at different sample concentrations. As displayed in Figure 8B, the Ibu-loaded sample did not show any considerable cell toxicity to the MDA-MB-231 cells. This might be due to the Ibu drug not being toxic to the tested cells. The MTT assay results suggest that the pNIPAm-co-pGMA-Mela hydrogel could be applied for pH- and temperature-responsive drug delivery applications. Furthermore, the blood compatibility behavior of the pNIPAm-co-pGMA-Mela hydrogel system was evaluated by hemolysis assay. As shown in Figure 8C, the pNIPAm-co-pGMA-Mela hydrogel-treated blood cells showed approximately 3.9–4.2% hemolysis compared to the positive control at different sample concentrations. These results confirm that the pNIPAm-co-pGMA-Mela hydrogel has a negligible hemolysis rate that is lower than the permissible range (5%) of hemolysis [39]. The observed result is similar to previously reported results in the literature [40,41]. These results further indicate that the developed pNIPAm-co-pGMA-Mela hydrogel is hemocompatible and has the potential to be an efficient drug delivery system for the administration of hydrophobic and/or hydrophilic drugs in drug delivery applications [42, 43].

**Figure 9:**
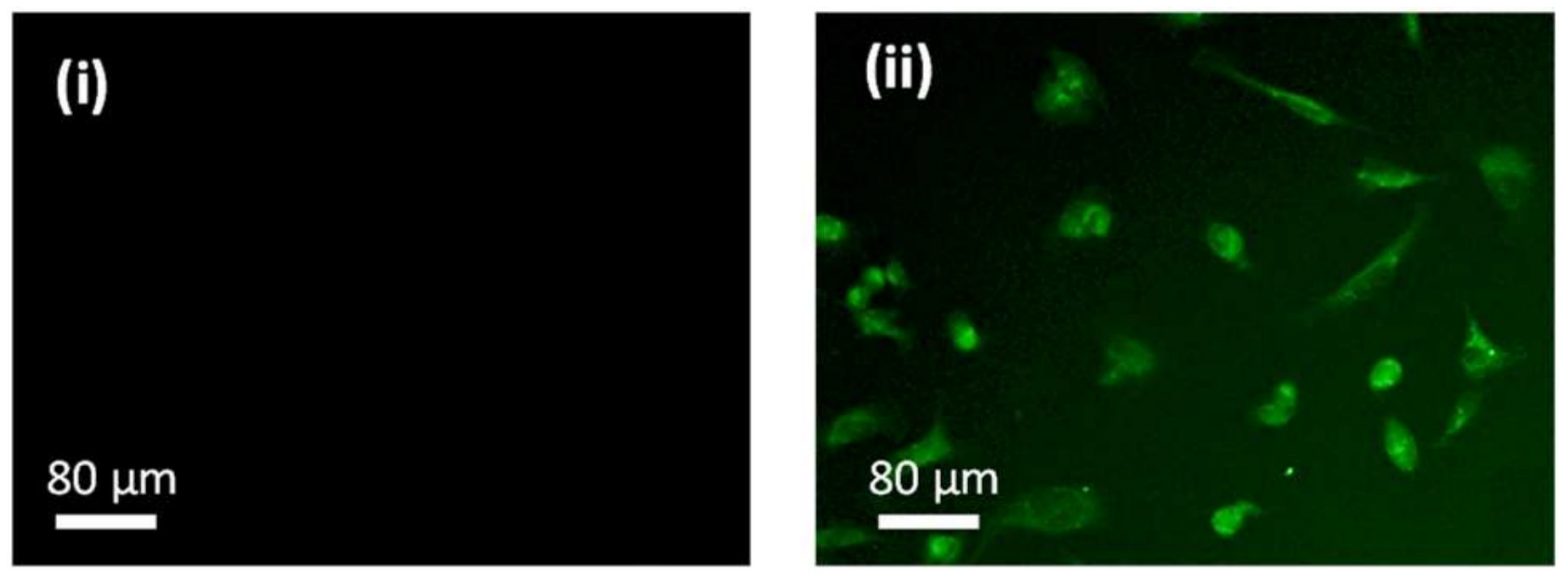
In vitro fluorescence cell images of (**i**) control without cells and (**ii**) MDA-MB-231 cells after treatment with pNIPAAm-co-pGMA-Mela hydrogel.

The in vitro cell viability of the pNIPAm-co-pGMA-Mela hydrogel was examined by exposing the samples to MDA-MB-231 cells at 37 °C under a 5% CO_2_ environment. As displayed in Figure 9, the prepared pNIPAm-co-pGMA-Mela hydrogel did not induce any cell death, suggesting that the pNIPAm-co-pGMA-Mela hydrogel is compatible with MDA-MB-231 cells.

## 3. Conclusions

In this work, we synthesized a melamine-functionalized pNIPAm-based copolymer hydrogel in the presence of pH and temperature stimuli for both hydrophobic and hydrophilic drug delivery applications. The drug loading and release behavior of the pNIPAm-co-pGMA-Mela hydrogel system was evaluated using Ibuprofen and 5-fluorouracil as model hydrophobic and hydrophilic drugs under different pH and temperature conditions. The pNIPAm segments and functionalized hydrophilic melamine groups in the pGMA segments of the pNIPAm-co-pGMA-Mela hydrogel can accommodate both hydrophobic and hydrophilic drugs. Our pH- and temperature-responsive drug delivery study results revealed that the pNIPAm-co-pGMA-Mela hydrogel system is responsive to both pH and temperature stimuli. The results showed that about ~100% of Ibu and 5-Fu were released at pH 4.0/45 °C. Moreover, the MTT assay and hemocompatibility analysis results proved that the pNIPAm-co-pGMA-Mela hydrogel system is biocompatible and hemocompatible, suggesting that it could be used for drug delivery applications. Overall, our experimental research results suggest that the pNIPAm-co-pGMA-Mela hydrogel system can be used in drug delivery applications for pH- and temperature-responsive administration of both hydrophobic and hydrophilic drugs.

## 4. Experimental Section

### 4.1. Reagents

2,2-azobisisobutyronitrile (AIBN, 12 wt.%), glycidyl methacrylate (GMA, 97%), N-Isopropylacrylamide (NIPAm, 97%), tetrahydrofuran (THF, 99%), diethyl ether (95%), Ibuprofen (98%), and 5-fluorouracil (99%) were purchased from Sigma Aldrich Chemical Co., St. Louis, MO, USA.

### 4.2. Synthesis of pNIAPAm-co-pGMA-Mela Copolymer Hydrogel

**Scheme 1:**
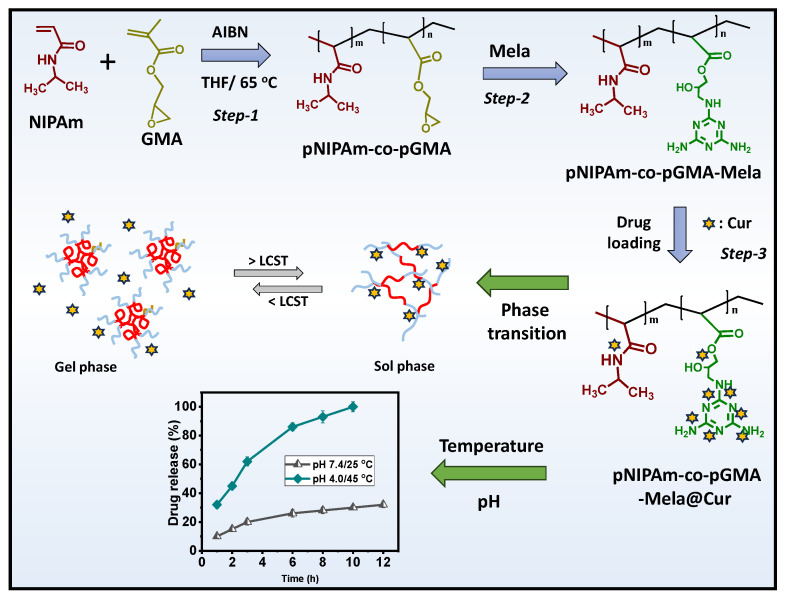
Illustration of the synthesis of pNIPAAm-co-pGMA copolymer (Step 1), melamine-functionalized pNIPAm-co-pGMA-Mela hydrogel (Step 2), and drug loading into the pNIPAAm-co-pGMA-Mela hydrogel system (Step 3).

For the typical synthesis, approximately 2 g (17.7 mmol) of NIPAm monomer was placed into a 50 mL three-necked flask containing 30 mL of THF solvent and equipped with N_2_ purging. To this end, about 2 g (17.7 mmol) of GMA was injected and stirred for 5 min. Then, the AIBN initiator (0.1 g) was added and allowed to react at 65 °C for 20 h. The obtained thick mass was poured into a 250 mL beaker containing diethyl ether and the obtained precipitate was dried at room temperature overnight [40]. The product was named pNIPAm-co-pGMA copolymer (Scheme 1, Step-1). Next, the melamine group was functionalized with pNIPAm-co-pGMA copolymer by reacting the amine groups of melamine with the epoxy groups of pGMA segments. For this functionalization process, about 0.5 g of pNIPAm-co-pGMA copolymer was dissolved in a reaction flask containing methanol (50 mL). To this end, 0.35 g (2.3 mmol) of melamine in methanol (5 mL) was added and magnetically stirred at 50 °C for 24 h [41,42,43]. After the reaction was completed, the obtained mixture was precipitated in diethyl ether and dried at 50 °C. The product was named pNIPAm-co-pGMA-Mela copolymer hydrogel (Scheme 1, Step 2).

### 4.3. Characterizations

A nuclear magnetic resonance (^1^H-NMR) instrument (OXFORD, 600 MHz, Concord, MA, USA) was used to confirm the synthesized pNIPAm-co-pGMA and pNIPAm-co-pGMA-Mela copolymer hydrogels. The surface morphology was measured using scanning electron microscopy (SEM, JEOL 6400, 10 kV, North Billerica, MA, USA). Fourier-transform infrared (FTIR) analysis was performed using a Perkin Elmer (Perkin Elmer, L1280138, Easton, MD, USA). Zeta potential analysis was performed using a Malvern Zetasizer (Nano-ZS, Malvern, UK) in a quartz cuvette; three repetitions were used obtain the average value. Acetic and basic buffers were used to control the pH of the solution media.

### 4.4. Turbidity Study

Turbidity measurement of the pNIPAm-co-pGMA-Mela copolymer hydrogel system was carried out by dissolving the copolymer sample (25 mg/mL) in 5 mL deionized water. Using a UV–visible spectrophotometer, the absorbance of the pNIPAm-co-pGMA-Mela copolymer hydrogel solution was measured at two different solution temperatures (25 °C and 45 °C).

### 4.5. Drug Loading and pH and Temperature-Stimuli-Responsive Release Study

Ibuprofen (Ibu) and 5-fluorouracil (5-Fu) were chosen as model hydrophobic and hydrophilic drugs, respectively. Approximately 10:5 wt:wt pNIPAm-co-pGMA-Mela copolymer:drug was taken for drug loading via diffusion under the swelling condition. To perform the drug loading, about 0.3 g of pNIPAm-co-pGMA-Mela copolymer sample was dissolved in 25 mL of deionized water. To this end, 5 mL of Ibu or 5-Fu (15 mg/mL) drug solution (methanol) was added and magnetically stirred for 12 h. The drug-loaded pNIPAm-co-pGMA-Mela copolymer was separated by heating the polymer solution to 60 °C, and the obtained supernatant solution was used to determine the amount of drug loaded into the pNIPAm-co-pGMA-Mela hydrogel sample by measuring the UV–vis absorption of Ibu at 272 nm and that of 5-Fu at 265 nm. The drug loading efficiency was determined as follows: drug loading efficiency (%) = [(Wt. of loaded drug)/(Wt. of feeding drug)] × 100. The calculated Ibu and 5-Fu loading efficiencies were determined to be about ~35% and ~47%, respectively.

The in vitro release of Ibu and 5-Fu from the drug-loaded pNIPAm-co-pGMA-Mela/Ibu and pNIPAm-co-pGMA-Mela/5-Fu hydrogel samples was assessed under two different pH conditions (physiological pH (pH 7.4) and acidic pH (pH 4.0)), three temperatures (25 °C, 37 °C, and 45 °C), and two pH + temperature combinations (pH 7.4/45 °C and pH 4.0/45 °C). For the drug release study, 50 mg of drug-loaded pNIPAm-co-pGMA-Mela/Ibu or pNIPAm-co-pGMA-Mela/5-Fu hydrogel sample was placed into a dialysis tube (MWCO 5000 kDa) and immersed in a beaker containing 25 mL phosphate buffer saline (PBS) media. The beaker was placed on a magnetic stirrer and gently stirred. The pH of the release medium was adjusted using the acidic or basic buffer and the temperature was maintained appropriately. At a predetermined time, about 1 mL of the release medium was taken out and the released drug was monitored using the UV–vis spectrometer at 272 nm for Ibu and 265 nm for 5-Fu. The amount of released drug was determined as follows: drug release (%) = [Mass of drug released at time t)/Total mass of drug in the sample] × 100.

### 4.6. MTT Assay Study

The in vitro biocompatibility of the pNIPAm-co-pGMA-Mela hydrogel without drug loading, pNIPAm-co-pGMA-Mela/5-Fu hydrogel, and pure 5-Fu drug were studied by 3-(4,5-dimethylthiazol-2-yl)-2,5-diphenyl tetrazolium bromide (MTT) assay analysis. MDA-MB-231 cells were seeded into a 96-well plate at 37 °C for 24 h. Then, the cells were exposed to pNIPAm-co-pGMA-Mela hydrogel dispersed in PBS buffer solution, pNIPAm-co-pGMA-Mela/5-Fu hydrogel, and pure 5-Fu samples and cultured for a further 5 h. Next, the MTT solution was added to each well and incubated for 4 h. Finally, the purple crystal was dissolved by adding 20 µL of cold dimethyl sulfoxide (DMSO) and the absorbance was determined using a microplate reader.

Cell viability (%) = [(OD_control_)/(OD_treated_)] × 100.

### 4.7. Hemocompatibility Study

We collected fresh mouse blood to conduct a standard hemolysis analysis following the published protocol [14,15,16]. The fresh blood was combined with an anticoagulant and mixed with sterilized physiological saline at a 4:5 ratio. Sterilized physiological saline served as the negative control, while distilled water served as the positive control. Subsequently, 0.2 mL of the extraction medium from the PEC:PHMG sample was incubated in a 37 °C water bath for 1 h. Following incubation, each tube was centrifuged and the absorbance of the supernatant was measured at a wavelength of 545 nm. The hemolysis ratio (HR) was calculated using the following formula:

HR = (Abs_positive control_ − Abs_negative control_)/(Abs_samples_ −Abs_negative control_),

where Abs is absorbance.

### 4.8. Statistical Analysis

The results were analyzed and expressed as the mean ± SD using a two-tailed Student’s *t*-test or one-way analysis of variance (ANOVA). The acceptable significance was *p* < 0.05.

## Data Availability

Data are contained within the article.
